# Prescribing of high-cost targeted therapies in England is diverging by region

**DOI:** 10.1016/j.puhip.2025.100717

**Published:** 2025-12-18

**Authors:** Julian Matthewman, Sinéad Langan, Reecha Sofat, James Galloway, Mark Russell

**Affiliations:** a London School of Hygiene & Tropical Medicine, Keppel St, London, WC1E 7HT, United Kingdom; b University of Liverpool, Foundation Building, Brownlow Hill, Liverpool, L69 7ZX, United Kingdom; c King's College London, Strand, London, WC2R 2LS, United Kingdom

**Keywords:** Targeted therapies, Biologics, Inflammatory disease, Integrated care boards, Secondary care medicines data, Regional variation

## Abstract

**Objectives:**

To examine regional variation in the prescribing of targeted therapies for chronic inflammatory disorders in England between 2019 and 2025.

**Study design:**

Retrospective observational study.

**Methods:**

This study analysed Secondary Care Medicines Data from all NHS hospitals in England to evaluate time-trends in prescribing rates of targeted therapies by Integrated Care Board (ICB).

**Results:**

Substantial and increasing regional variation in prescribing rates for targeted therapies was observed between 2019 and 2025. The disparity between the highest and lowest prescribing ICBs increased over time, with rates ranging from 2.0 to 6.5 per 1000 people in 2019 and 3.4 to 14.2 per 1000 people in 2025.

**Conclusions:**

There is marked and growing regional variation in the prescribing of targeted therapies across England. Further research should explore the reasons for this divergence to ensure equitable access to these highly effective treatments for patients with chronic inflammatory disorders, irrespective of geography.

## 1 Introduction

Targeted therapies, including biologics and small molecule therapies, have transformed outcomes for people with chronic inflammatory disorders, including rheumatoid arthritis, psoriasis, and inflammatory bowel disease [1]. Without prompt remission-directed therapy, chronic inflammatory conditions can result in substantial morbidity, for example irreversible joint damage, cutaneous scarring, or bowel perforation. Inadequate disease control can also lead to work disability and psychological morbidity, as well as potentially avoidable surgery (e.g. joint replacements or bowel resection). Timely and appropriate escalation to targeted therapies - often referred to as the “window of opportunity” - leads to better short- and long-term outcomes for patients [2]. The importance of targeted therapies is evidenced, for example, by the recent inclusion of targeted therapies for psoriasis in the World Health Organisation's list of essential medicines [3].

Using population-level data, previous studies have shown the number of people prescribed targeted therapies in England increased by 62 % between 2019 and 2024 (from 4.11 people to 6.64 people per 1000 population) [4]. While these national trends are encouraging, it potentially masks significant underlying variation in prescribing practices at a regional level. With high costs for targeted therapies, there are challenges to ensuring equitable prescribing.

We know from different countries and settings that prescribing of targeted therapies can be inequitable. Recent studies in the United Kingdom and the United States have found inequities in the prescribing of biologics by ethnicity, age, and income for rheumatoid arthritis [5], psoriasis [6], and inflammatory bowel disease [7]. For rheumatoid arthritis care in England, we also know that, despite a universal health-care system, there is marked variation in biologic prescribing across the seven broad administrative regions of the National Health Service (NHS) [8]. However, it remains poorly understood the extent to which this prescribing variation is evident at more granular geographical levels – particularly between Integrated Care Boards (ICBs), where commissioning of NHS care in England occurs.

The objective of this study was to examine regional variation in the prescribing of targeted therapies for chronic inflammatory disorders throughout ICBs in England between 2019 and 2025.

## 2 Methods

We performed a retrospective observational study to explore differences in prescribing of biologics and targeted therapies for inflammatory conditions by ICB in England. To do so, we utilised Secondary Care Medicines Data (SCMD) [9], which are published monthly by the NHS Business Services Authority. These data contain pharmacy stock control data for medications at Virtual Medicinal Product level (VMP; i.e. medication, strength and form), aggregated by NHS trust in England (all NHS Acute, Teaching, Specialist, Mental Health and Community Hospital Trusts) and by month. No individual-level data are available. We used finalised SCMD data (from April 2019 to March 2025) and provisional data (from April 2025 to September 2025). We extracted information on the issued quantities of therapies targeting BAFF, CD20, CTLA-4, IL-1, IL-12/23, IL-17, IL-4/13, IL-5, IL-6, IgE, Integrin, JAK, PDE4, S1P, and TNF. Product SNOMED codes and further details for each drug were previously described [1].

We calculated Defined Daily Dose (the average maintenance dose per day for a drug used for its main indication in adults) using WHO definitions [10], with modifications based on clinical knowledge.(Supplement of reference 4) To estimate the number of people prescribed each drug, we divided monthly defined daily doses by 30.4 (i.e. the mean number of days in a month, averaged across the year). We excluded 11 outliers for Rituximab from the University Hospitals Plymouth NHS Trust and 1 outlier for Ustekinumab from the East Lancashire Hospitals NHS Trust, where the Defined Daily Dose was larger than 100 times the interquartile range.

To estimate the number of people prescribed a targeted therapy per 1000 population we used mid-year population estimates for Integrated Care Boards from the Office of National Statistics for 2019 to 2022 [11], and projected population estimates for 2023 to 2025 (we estimated mid-year population estimates for 2023 to 2025 by fitting a linear model to the data from 2019 to 2022 and predicting values for 2023 to 2025).

## 3 Results

**Fig. 1:**
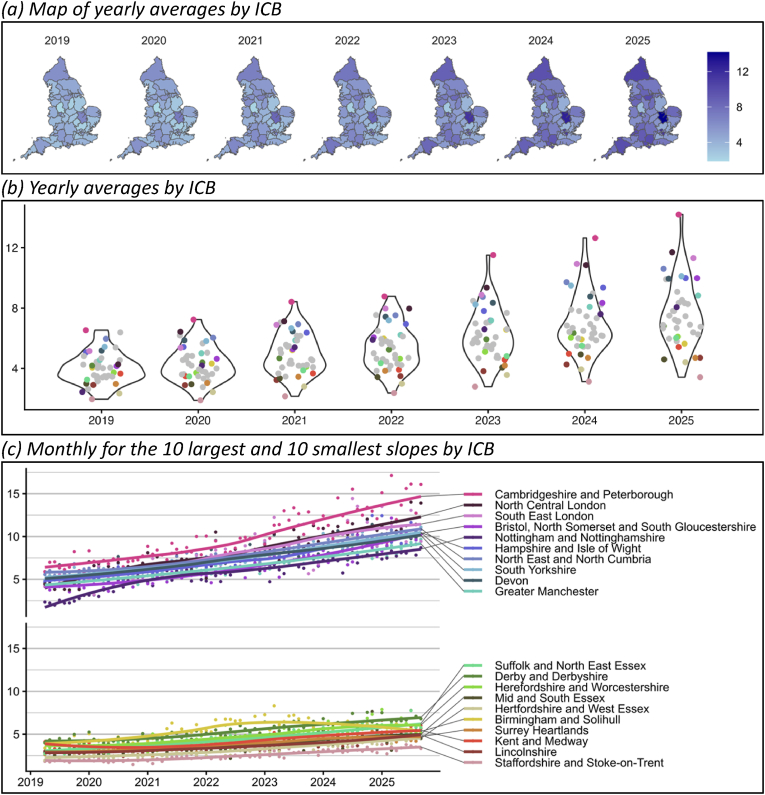
Number of people prescribed targeted therapies per 1000 population.

Overall, the number of people prescribed targeted therapies is increasing across England but has been diverging by ICB since 2019 (Fig. 1). In 2019, the number of people prescribed targeted therapies ranged from 2.0 to 6.5 per 1000 people. In 2025, the range was 3.4–14.2 per 1000 people. ICBs with the most prescribing in 2025 were Cambridgeshire and Peterborough (14.2 per 1000), North Central London (11.7 per 1000), and South East London (11.3 per 1000). ICBs with the least prescribing in 2025 were Staffordshire and Stoke on Trent (3.4 per 1000), Hertfordshire and West Essex (4.4 per 1000), and Mid and South Essex (4.6 per 1000). Some ICBs with low prescribing in 2019 had large increases by 2025 (e.g. Nottingham and Nottinghamshire from 2.4 per 1000 to 8.1 per 1000), while others have remained at a low level (e.g., Staffordshire and Stoke-on-Trent from 2.0 per 1000 to 3.4 per 1000). Yearly averages for all ICBs are shown in Fig. 1.

## 4 Discussion

Our findings point to substantial regional variation in the prescribing of targeted therapies throughout England, with increasing divergence in prescribing rates over time. Despite having a universal healthcare system with national prescribing guidelines (e.g., from the National Institute for Health and Care Excellence), we showed that some ICBs have remained persistently low prescribers of targeted therapies, while others have increased markedly over the past 6 years. Given the impact targeted therapies can have on patient outcomes, highlighting this variation is an important first step to motivate further research and ultimately change policy and practice to ensure equitable treatment.

There are many potential reasons for the observed divergence between ICBs. These could include differences in commissioning policies [12], variation in treatment pathways [13], budgetary constraints, rates of biosimilar uptake, population health needs, NHS Trust catchment areas, the availability of specialist services, and patient- and doctor-level decisions. These factors must be explored further if we are to ensure equitable access to highly effective treatments for chronic inflammatory disorders, irrespective of geography.

Our findings add to the existing evidence demonstrating inequitable prescribing of targeted therapies by factors including age, ethnicity and income; findings which have been demonstrated across multiple chronic inflammatory conditions and throughout different healthcare systems worldwide [[5], [6], [7]]. While the NHS has a universal healthcare system, our findings suggest that there may be worsening inequity at a regional level where commissioning decisions are made.

Importantly, our findings also demonstrate how population-level medicines data can be used to address questions on prescribing equity, with transferable learnings for other healthcare systems worldwide. Using these data more routinely (e.g. through new platforms such as OpenPrescribing Hospitals) has enormous potential to transform how prescribing patterns are monitored in near real-time and enable resources to be targeted towards reducing inequity [14].

## Author contributions

JM contributed to Conceptualization, Data curation, Formal analysis, Investigation, Methodology, Project administration, Resources, Validation, Visualization, Writing – original draft, Writing – review & editing.

SL contributed to Conceptualization, Funding acquisition, Supervision, Writing – review & editing.

RS contributed to Validation, Writing – review & editing.

JG contributed to Conceptualization, Data curation, Supervision, Validation, Writing – review & editing.

MR contributed to Conceptualization, Data curation, Investigation, Methodology, Resources, Supervision, Validation, Writing – original draft, Writing – review & editing.

## Ethical statement

This work is based on fully open-source aggregate-level data. Therefore, no ethical approval is required.

## Funding

This work was supported by 10.13039/501100023699Health Data Research UK, an initiative funded by 10.13039/100014013UK Research and Innovation, 10.13039/501100000276Department of Health and Social Care (England) and the devolved administrations, and leading medical research charities.

## Declaration of competing interest

JG has received honoraria from Abbvie, Biovitrum, BMS, Celgene, Chugai, Galapagos, Gilead, Janssen, Lilly, Novartis, Pfizer, Roche, Sanofi, Sobi and UCB; and grant funding from Sandoz UK. MR has received honoraria from AbbVie, Biogen, Galapagos, Johnson & Johnson, Lilly, Menarini, Novartis, Pfizer, UCB and Viforpharma; and grant funding from Sandoz UK.
